# Emotional disorders, stress and ways of coping with them among the adult population of Russia during the first wave of the COVID-19 pandemic

**DOI:** 10.1192/j.eurpsy.2026.11013

**Published:** 2026-07-31

**Authors:** V. I. Rozhdestvenskiy, V. V. Titova, I. A. Gorkovaya, D. O. Ivanov, Y. S. Aleksandrovich

**Affiliations:** Department of Psychosomatics and Psychotherapy, Saint Petersburg State Pediatric Medical University, Saint Petersburg, Russian Federation

## Abstract

**Introduction:**

During the first wave of the COVID-19 pandemic, Russian adults not directly involved in the medical field experienced affective disorders, primarily depression and anxiety, along with elevated stress levels, largely triggered by objective external factors. To manage these symptoms, individuals employed a range of coping strategies, which varied in their effectiveness.

**Objectives:**

The study aimed to assess the severity of emotional disorders (depression and anxiety) and stress among adult Russian citizens not directly involved in the medical field during the first wave of the COVID-19 pandemic. In addition, the relationships between emotional disorders, stress, and coping strategies were examined.

**Methods:**

Data were collected between May and July 2020 using a Google Form developed by the authors. The sample consisted of 97 adult participants residing in Russia. Coping strategies were assessed with the COPE-60 inventory, and symptoms of depression, anxiety, and stress were measured using the DASS-21 scale. Both instruments had been previously adapted for use in the Russian context.

**Results:**

We found that 80% of respondents had no symptoms of depression, 93% had no symptoms of anxiety, and 89% had no symptoms of stress. A moderate degree of depression was diagnosed in 14% of respondents, a moderate degree of anxiety in 1% of respondents, and a moderate degree of stress in 8%. Both severe depression and severe anxiety were observed in 6% of respondents, and severe stress in only 3%. No statistically significant correlations were found between depression and coping strategies. Anxiety had negative correlations with the coping strategies ‘active coping’ (r_s_ = -0.215, p < 0.05) and ‘planning’ (r_s_ = -0.249, p < 0.05). In contrast, stress had negative correlations with ‘active coping’ (r_s_ = -0.254, p < 0.05), ‘suppression of competing activities’ (r_s_ = -0.201, p < 0.05) and ‘planning’ (r_s_ = -0.209, p < 0.05).

**Conclusions:**

During the first wave of the COVID-19 pandemic in Russia, most adults who were not directly involved in medicine did not experience depression, anxiety or stress. The coping strategies used by Russians were not effective in combating depression. At the same time, the most productive coping strategies for overcoming anxiety and stress were those based on active attempts to change the situation in which the respondents found themselves. However, focusing exclusively on the problem itself only reduced the level of stress and did not reduce anxiety.

**Disclosure of Interest:**

None Declared

